# Comparative Evaluation of Serum and Gingival Crevicular Fluid Periostin Levels in Periodontal Health and Disease: A Biochemical Study

**DOI:** 10.7759/cureus.7218

**Published:** 2020-03-09

**Authors:** Khumukcham Sophia, Snophia Suresh, Uma Sudhakar, Shaik Abdul Cader, Varsha M Vardhini, Lalitha T Arunachalam, S. Catherine Jean

**Affiliations:** 1 Periodontics, Jawaharlal Nehru Institute of Medical Sciences, Imphal, IND; 2 Periodontics, Thai Moogambigai Dental College & Hospital, Chennai, IND

**Keywords:** chronic periodontitis, gingivitis, periostin, gingival crevicular fluid, biomarker, serum

## Abstract

Introduction

Periostin, a secreted adhesion molecule, is a matricellular protein secreted most in periodontal ligament and periosteum. This periostin is needed for integrity and maturation of periodontal tissue. The present study was conducted to estimate and compare the gingival crevicular fluid and serum periostin levels in subjects having chronic periodontitis, gingivitis and healthy periodontium.

Methods

Ninety patients belonging to both sexes were categorized into three groups, 30 patients each as healthy periodontium (Group I), chronic gingivitis (Group II) and generalised chronic periodontitis (Group III). The clinical parameters included assessment of plaque index (PI), gingival index (GI), probing pocket depth (PPD) and clinical attachment level (CAL). Gingival crevicular fluid (GCF) and serum samples were collected and the enzyme-linked immunosorbent assay was used to estimate periostin levels.

Results

Periostin levels in GCF were comparatively low in the chronic periodontitis than in the gingivitis and healthy periodontium groups and the difference was statistically significant. No statistical difference was found for serum periostin levels among Group I, Group II and Group III. On comparison of clinical parameters, significant difference was noticed among the three groups. GCF periostin levels were correlated inversely with the clinical parameters in chronic periodontitis patients.

Conclusion

GCF periostin levels were gradually reduced with the increase in severity of periodontal disease. This novel biomarker has role in maintaining normal periodontal tissue function and may be used as a potential marker in periodontal disease activity evaluation.

## Introduction

Periodontal disease involves the interaction of the biofilm and host immune-inflammatory response, affects the integrity of the periodontal structures, resulting in destruction of connective tissue and resorption of alveolar bone [1]. Gingivitis is characterized by inflammation that is confined to the gingiva, without loss of periodontal attachment apparatus. The host’s immune inflammatory response is markedly different in individuals who develop periodontitis compared to individuals who never progress beyond gingivitis. The components of gingival crevicular fluid (GCF) have commonly been considered to find out the individuals with periodontal disease activity [2].

Periostin is an 811 amino acid protein, originally identified in murine osteoblasts. Periostin showed structural similarity with fasciclin-1, which is an insect axonal guidance protein and it is also termed as osteoblast specific factor-2 [3]. Periostin derived its name, because of its expression in periodontal ligament and periosteum [4]. It is an extracellular matrix protein, has a role in connective tissue integrity and cell migration and adhesion. Another important role of periostin is wound repair, causes interaction of type I collagen with fibronectin, thereby helps in remodelling the periodontium [5]. Periostin is secreted by connective tissues rich in collagen, releases periostin when they are subjected to mechanical stresses [6]. Periostin is also associated with tooth eruption processes promoting adhesion and migration of various cell types, leading to formation of mineralized tissues of the tooth and periodontium [7]. Periostin is entrapped between the cytoplasmic processes of periodontal fibroblasts, cementoblasts and also the surrounding collagen fibrils [8]. Hence periostin may be used as a periodontal regeneration marker and the GCF periostin levels have been found to reduce with the increase of the severity of periodontal disease [9]. Since limited studies are available to find out the role of periostin in periodontal disease, our study was aimed to compare and evaluate the serum periostin and gingival crevicular fluid periostin levels in periodontally healthy, gingivitis and chronic periodontitis subjects.

## Materials and methods

Ninety subjects belonging to both sexes were selected from the outpatient clinics of the department of periodontics. Subjects were divided into three groups of 30 subjects each as healthy periodontium (Group I), chronic gingivitis (Group II) and generalised chronic periodontitis (Group III).

The inclusion criteria for healthy periodontium (Group I) were subjects having good oral hygiene, no bleeding on probing, no visual signs of gingival inflammation, the gingival Index score of “0”, probing pocket depth and clinical attachment level of ≤3 mm. The inclusion criteria for chronic gingivitis (Group II) were presence of visual inflammatory signs, gingival index ≥ 2, probing pocket depth and clinical attachment level ≤ 3 mm and the inclusion criteria for generalised chronic periodontitis (Group III) were presence of inflammatory changes in periodontal tissues, gingival index ≥ 2, probing pocket depth and clinical attachment level ≥ 4 mm in >30% sites involving minimum of eight teeth and evidence of radiographic bone loss [10]. Patients having aggressive forms of periodontal disease, history of periodontal treatment received in the past six months, underlying systemic diseases, patients on high dose steroid therapy, pregnancy, lactation and smokers were excluded.

**Figure 1:**
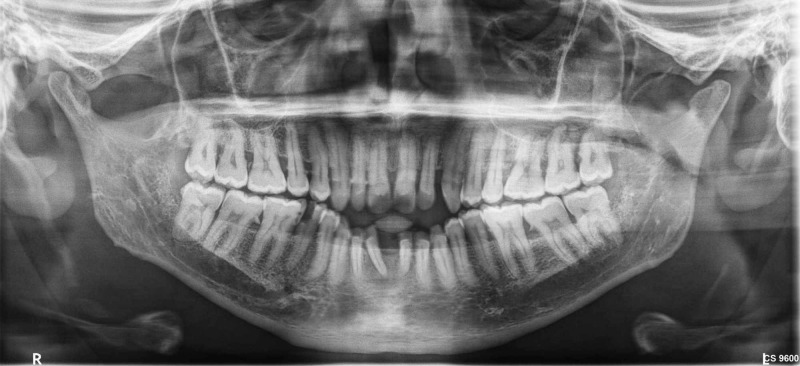
Orthopantomogram of a chronic periodontitis patient

Ethical committee approval was obtained (Dr.MGRDU/TMDCH/RES/2016/2582), the purpose and type of the study was verbally explained to the subjects and written consent was taken. The clinical periodontal parameters including, plaque index (PI), gingival index (GI), probing pocket depth (PPD) and clinical attachment level (CAL) were evaluated for the subjects. Midbuccal, distobuccal, mesiobuccal and palatal sites in each tooth were recorded for PI [11]. The buccal, mesial, distal and lingual gingival areas were examined for GI [12]. PPD and CAL were measured in millimetres and were assessed in all the teeth at six sites and CAL was calculated from cemento-enamel junction to periodontal pocket base [13,14]. Orthopantomogram (OPG) was taken for each patient and the bone loss (Figure 1) in the OPG was determined to identify chronic periodontitis patients.

GCF and serum periostin analysis

Supra gingival scaling was done one day before collection of GCF. A volume of 1 (microlitre) was procured from each test site by an extracrevicular approach, using volumetric microcapillary pipettes. The collected GCF was transferred immediately to Eppendorf tubes and venous samples (2 ml) were collected from the antecubital vein, and it was transferred to clot activator coated tube and centrifuged at 3000 x g for 5 minutes. The GCF and serum samples were stored at -70°C until the time of assay. The periostin levels were estimated using Enzyme-Linked Immunosorbent Assay (ELISA). It consists of 96 well plates coated with human periostin specific antibody. Standards and sample solution were added to the wells, periostin present in the sample binds to the antibody. Biotinylated anti-human periostin antibody was poured and by washing unbound biotinylated antibody was removed. Then horseradish peroxide conjugated streptavidin was poured, followed by tetramethyl benzidine solution into the wells. Blue colour was observed, which after addition of stop solution was converted to yellow. The colour intensity was correlated with the periostin levels and it was read in ELISA reader. The periostin levels were expressed in ng/ml.

Statistical analysis

The SPSS software program version 22 (IBM Corp., Armonk, NY) was used to perform statistical analysis. Kruskal-Wallis test was applied to assess the mean difference in the clinical parameters and periostin levels in GCF and serum among Group 1, Group II and Group III. Spearman’s rank correlation co-efficient test was used to test the correlation between GCF periostin with the clinical parameters. Level of significance was fixed at p < 0.05.

## Results

The clinical parameters (PI, GI, PPD and CAL) were compared among the groups (Table 1). On comparison of the mean PI, GI, PPD and CAL, the difference was found to be significant and these levels were lowest in Group I and highest among Group III. GCF periostin and serum periostin were compared among the groups and the mean GCF periostin levels were lowest in Group III and highest in Group I (Table 2). Comparison of serum periostin levels among the three groups, did not show statistically significant difference. Negative correlation was observed on correlating GCF periostin levels with clinical parameters (Table 3).

**Table 1:** Comparison of mean clinical parameters among three groups *Level of significance: P < 0.05 significant PI: Plaque index; GI: Gingival index; PPD: Probing pocket depth; CAL: Clinical attachment level; SD: Standard deviation; P-value: probability value.

Parameters	Group	Mean ± SD	Mean Rank	p-value
PI	Group I	0.30 ± 0.06	13.00	0.001*
	Group II	1.20 ± 0.23	44.36	
	Group III	1.96 ± 0.32	56.64	
GI	Group I	0.10 ± 0.37	13.00	0.001*
	Group II	2.60 ± 0.20	58.30	
	Group III	2.30 ± 0.28	42.70	
PPD (mm)	Group I	2.02 ± 0.28	14.92	0.001*
	Group II	2.46 ± 0.29	36.08	
	Group III	5.56 ± 0.52	63.00	
CAL (mm)	Group I	1.51 ± 0.32	15.33	0.001*
	Group II	2.06 ± 0.25	34.28	
	Group III	5.32 ± 0.53	62.00	

**Table 2:** Comparison of mean GCF and serum periostin levels among three groups *Level of significance: P < 0.05 significant. GCF: Gingival crevicular fluid; P-value: Probability value.

Parameters	Group	Mean ± SD	Mean Rank	p-value
GCF PERIOSTIN (ng/ml)	Group I	24.77 ± 4.46	56.64	0.001*
	Group II	21.46 ± 2.97	42.00	
	Group III	15.86 ± 2.85	15.36	
Serum Resistin (ng/ml)	Group I	38.4 ± 13.10	51.26	0.112
	Group II	37.7 ± 8.63s	49.02	
	Group III	35.87 ± 6.80	43.72	

**Table 3:** Correlation of GCF periostin levels with the clinical parameters and serum periostin levels *Level of significance: P < 0.05 significant. PI: Plaque index; GI: Gingival index; PPD: Probing pocket depth; CAL: Clinical attachment level; SD: Standard deviation; P-value: Probability value; r: Correlation.

Groups		PI	GI	PPD	CAL	SERUM
Group I	r	0.226	-0.513*	0.183	-0.158	0.517
	p	0.278	0.03	0.382	0.460	0.02*
Group II	r	-0.334	-0.509*	-0.100	-0.027	0.522
	p	0.103	0.004	0.635	0.899	0.02*
Group III	r	-0.197	-0.485*	-0.984^**^	-0.969^**^	0.461
	p	0.258	0.01	0.000	0.000	0.02*

## Discussion

Periodontitis is initiated by tooth-associated microorganisms organized as a biofilm, and evokes a host inflammatory response. Reversible inflammatory changes occur in gingivitis, but non-reversible destruction happens in periodontitis and if not treated leads to tooth loss [15]. Periostin being a multifaceted protein, has been involved in repair and regenerative processes of various tissues. It is also a matricellular modulator and it is considered as a biomarker for various diseases [16]. The 90 and 87-kDa isoforms are produced by neuroectodermal-derived fibroblasts [17]. Once it is secreted, periostin has an affinity to bind to molecules such as tenascin-C, collagen and BMP-1 [18]. This property helps in the extracellular matrix maturation and increases strength of the tissue [19]. Periostin also possesses potent mitogenic properties and binds with the cell membrane via integrins [20]. Periostin shows greater specificity, among the proteins expressed in periodontal ligament. The purpose of the current study was to investigate GCF and serum periostin levels in individuals with clinically healthy periodontium, gingivitis and chronic periodontitis patients.

In the present study, the mean PI was comparatively low in Group I than in Group II and Group III which showed a statistically significant difference. The mean scores of GI were higher in Group III than in Group I. This finding was comparable to the previous study done by Bhardwaj et al. where they observed that the mean PI and GI scores were high in chronic periodontitis compared to healthy patients [21]. Plaque is the primary disease-initiating factor that resulted in transition from health to gingivitis, and if left untreated gingivitis might progress to periodontitis [22].

In our study, the mean PPD was comparatively high in Group III than in Group II and Group I and the difference was statistically significant. The mean CAL was higher in Group III than in Group II and Group I which showed a statistically significant difference. The results of the present study are comparable to the study done by Pradeep et al. where they found mean PPD and CAL in chronic periodontitis was higher than in the gingivitis and healthy patients [23]. The microorganisms from supra- and subgingival plaque are needed for the development of gingivitis and periodontitis. Periodontal disease is mostly a plaque-induced disease that starts as gingivitis, affecting the adult populations with the age of 35 to 40 years. Once the disease was initiated, without treatment, a slow progressive destruction will be observed [24].

The mean level of GCF periostin was low in the patients with Group III when compared to Group II and Group I and the mean difference was statistically significant. This study was in accordance with the previous studies which reported that GCF periostin level in chronic periodontitis was lowest when compared to healthy periodontium [9,25]. Decrease in GCF periostin levels was observed proportionally with periodontal disease progression and the degree of inflammation had affected the GCF periostin levels strongly and negatively. Decreased level of GCF periostin in periodontitis might be considered that this molecule has role in maintaining the function of normal periodontal tissue. Bacterial competition and the reduction in periodontal ligament fibroblasts numbers in periodontitis subjects, would have reduced GCF periostin levels. Reduction in GCF periostin levels may compromise the periodontal ligament stability and exacerbate the inflammatory process due to reduction in structural and biochemical potential of periodontal ligament [6,26].

Aral et al. have also observed the reduction in GCF periostin levels in chronic periodontitis and aggressive periodontitis patients and this delay in tissue repair associated with the periodontal tissues inflammation, would have reduced the periostin levels and accelerates the disease progression [27]. A previous study also reported that an increase in GCF periostin levels was observed following periodontal surgery and this increase is due to reduction of chronic inflammatory stimuli following surgical procedure [28]. Esfahrood et al. observed significant relationship between salivary periostin levels and chronic periodontitis [29].

Comparison of the mean serum periostin levels among the groups showed no statistically significant difference. This finding was comparable to the previous study by Balli et al., and also reported no significant difference in serum periostin levels among the groups [9]. Local and low-grade infection associated with periodontitis might not have affected the serum periostin levels.

GCF periostin levels were associated with clinical parameters in respect to GI, PPD and CAL which was comparable to previous studies, which reported that the periostin level is decreased by P. gingivalis lipopolysaccharides and tumor necrosis factor-α in periodontal ligament fibroblasts, which accepts the fact that the inflammatory process negatively influenced the periostin levels [30].

Our study being an observational study proves the association of GCF periostin levels with periodontal disease. Future longitudinal studies involving more participants with interventional trial may avoid these limitations.

## Conclusions

In our study GCF periostin levels were reduced with the increase in periodontal disease severity and these GCF periostin levels were inversely correlated with the clinical periodontal parameters. The present finding may support the concept that periostin levels increase, may lead to rapid tissue repair and gain in periodontal attachment. Within the limits of the study, it may be accepted that the GCF periostin level can be considered as a potential biomarker in periodontal disease activity evaluation.
